# Regulation of SCN3B/scn3b by Interleukin 2 (IL-2): IL-2 modulates *SCN3B/scn3b* transcript expression and increases sodium current in myocardial cells

**DOI:** 10.1186/s12872-015-0179-x

**Published:** 2016-01-04

**Authors:** Yuanyuan Zhao, Qiaobing Sun, Zhipeng Zeng, Qianqian Li, Shiyuan Zhou, Mengchen Zhou, Yumei Xue, Xiang Cheng, Yunlong Xia, Qing Wang, Xin Tu

**Affiliations:** Key Laboratory of Molecular Biophysics of Ministry of Education, College of Life Science and Technology, Center for Human Genome Research, Cardio-X Institute, Huazhong University of Science and Technology, Wuhan, 430074 China; First Affiliated Hospital of Dalian Medical University, Dalian, 116011 China; The Laboratory of Cardiovascular Immunology, Institute of Cardiology, Union Hospital, Tongji Medical College of Huazhong University of Science and Technology, Wuhan, 430074 China; Henan Research Institute of Population and Family Planning, National Health and Family Planning Commission Key Laboratory of Birth Defects Prevention, Zhengzhou, 450002 China; Department of Cardiology, Guangdong General Hospital, Guangdong, 510030 China

**Keywords:** Interleukin-2, *SCN3B*, Sodium current density, *p53*

## Abstract

**Background:**

In the initiation and maintenance of arrhythmia, inflammatory processes play an important role. IL-2 is a pro-inflammatory factor which is associated with the morbidity of arrhythmias, however, how IL-2 affects the cardiac electrophysiology is still unknown.

**Methods:**

In the present study, we observed the effect of IL-2 by qRT-PCR on the transcription of ion channel genes including *SCN2A*, *SCN3A*, *SCN4A*, *SCN5A*, *SCN9A*, *SCN10A*, *SCN1B*, *SCN2B*, *SCN3B*, *KCNN1*, *KCNJ5*, *KCNE1, KCNE2*, *KCNE3*, *KCND3, KCNQ1, KCNA5, KCNH2* and *CACNA1C*. Western blot assays and electrophysiological studies were performed to demonstrate the effect of IL-2 on the translation of *SCN3B/scn3b* and sodium currents.

**Results:**

The results showed that transcriptional level of *SCN3B* was up-regulated significantly in Hela cells (3.28-fold, *p* = 0.022 compared with the control group). Consistent results were verified in HL-1 cells (3.73-fold, *p* = 0.012 compared with the control group). The result of electrophysiological studies showed that sodium current density increased significantly in cells which treated by IL-2 and the effect of IL-2 on sodium currents was independent of *SCN3B* (1.4 folds, *p* = 0.023). Western blot analysis showed IL-2 lead to the significantly increasing of *p53* and *scn3b* (2.1 folds, *p* = 0.021 for *p53*; 3.1 folds, *p* = 0.023 for *scn3b*) in HL-1 cells. Consistent results were showed in HEK293 cells using qRT-PCR analysis (1.43 folds for *P53*, *p* = 0.022; 1.57 folds for *SCN3B*, *p* = 0.05).

**Conclusions:**

The present study suggested that IL-2, may play role in the arrhythmia by regulating the expression of *SCN3B* and sodium current density.

**Electronic supplementary material:**

The online version of this article (doi:10.1186/s12872-015-0179-x) contains supplementary material, which is available to authorized users.

## Background

In the initiation and maintenance of arrhythmia, inflammatory processes play an important role. Clinic studies have observed that pro-inflammatory factors such as tumor necrosis factor α (TNF-α), Interleukin 1 (IL-1) family members [1] and monocyte chemo attractant protein 1 (MCP-1) were associated with arrhythmia [2]. Basic studies also revealed that pro-inflammatory factors could regulate the expression of ion channel genes and induced abnormal cardiac electrophysiological activity [3, 4].

IL-2 is a pro-inflammatory factor which can induce T-cell proliferation, regulate the expression of Na/K pump in human lymphocytes [5] and participate in inflammatory processes. IL-2 has been reported to be associated with various cardiac arrhythmias including atrial fibrillation (AF) and ventricular tachycardia (VT). Ioannis Rizos *et al.* found that low serum IL-2 was associated with hypertension and/or chronic stable coronary artery disease and recent onset AF [6]. Lukasz Hak *et al.* observed that high serum level concentration of IL-2 might be a predictive factor for early postoperative AF in cardiopulmonary bypass graft (CABG) patients [7]. Although studies observed that IL-2 was associated with the morbidity of arrhythmias, however, how IL-2 affects the cardiac electrophysiology is still unknown.

In the present study, we hypothesized that IL-2 might affect ion channels directly as a pro-inflammatory cytokine, and observed the effect of IL-2 on the expression of ion channel genes including *SCN2A*, *SCN3A*, *SCN4A*, *SCN5A*, *SCN9A*, *SCN10A*, *SCN1B*, *SCN2B*, *SCN3B*, *KCNN1*, *KCNJ5*, *KCNE1, KCNE2*, *KCNE3*, *KCND3, KCNQ1, KCNA5, KCNH2* and *CACNA1C*[8].

## Methods

### Cell lines and plasmids

Cell lines HEK293 (Human embryonic kidney cell), HeLa (Human cervical carcinoma cell) and mouse myocardial HL-1 cells were used in the study ((American Type Culture Collection, Rockville, MD, USA).

HEK293 and HeLa cells were cultured in the Dulbecco’s Modified Eagle’s medium (DMEM) and supplemented with 10 % fetal bovine serum (FBS, Gibco Life Technologies, Gaithersburg, MD, USA) in the humidified incubator with 5 % CO_2_ at 37 °C. For HL-1 cells, tissue culture flasks were coated with 0.02 % gelatin and 5 μg/ml fibronectin (Gibco Life Technologies, Gaithersburg, MD, USA) and incubated at 37 °C overnight. Then gelatin/fibronectin was removed, and HL-1 cells were cultured in the flasks. HL-1 cells were cultured in Claycomb medium (Gibco Life Technologies, Gaithersburg, MD, USA) and supplemented with 2 mM L-glutamine, 0.1 mM norepinephrine,100U/ml penicillin, 100 μg/ml streptomycin and 10 % FBS (Gibco Life Technologies, Gaithersburg, MD, USA) in the humidified incubator with 5 % CO_2_ at 37 °C.

Cultured cells were treated by IL-2 [IL-2 treated group: 1 ng/μl IL-2 (PeproTech, New Jersey, USA) was diluted into 100 ng/μl by ddH_2_O)] or ddH_2_O (control group) respectively when cells were cultured for 70-80 % confluent in plates.

The full length cDNA of *SCN5A* (GenBank: NC_000003.11) and *SCN3B* (GenBank: NC_000011.9) were amplified using human genomic cDNA and cloned them into plasmid of pcDNA 3.0 and pEGFP-N1 (pcDNA-SCN5A and pEGFP-N1-SCN3B) respectively.

### Quantitative real time PCR analysis

Cells were harvested after 48 h and then lysed by using RNAiso plus (TaKaRa, Dalian, China). Total RNA was isolated from cells and converted into cDNA by reverse transcription with the First-Strand cDNA Synthesis kit (Toyobo, Japan) using OligodT.

qRT-PCR analysis was carried out using FastStart Universal SYBR Green Master kit (Roche Applied Science, Mannhein, Germany) with 10 μl reaction volume on ABI 7900 Genome Analyzer System. The reaction system was 2 μl cDNA template, 5 μl SYBR green (including ROX) mix, 200nM forward and reverse primers. Human gene *GAPDH* was used as internal standard for HEK293 and HeLa cells, mouse gene *Gapdh* was used as internal standard for HL-1 cells. The primers for RT-qPCR analysis were listed in Table 1. The PCR products were verified by melting curve analysis and the results were analyzed using 2^-ΔΔCt^ method as described [9]. Each examination was performed in triplicated and repeated at least three times.

**Table 1:** The primers of cDNA of ion channels related genes and *GAPDH/Gapdh* for RT-PCR analysis

Gene	F	R
*SCN3B(human)*	attgtttcccctggcttctc	gcctccacctcctctctctt
*Scn3b(mouse)*	catcctcctggtcttcctcac	cgggtaccacagagttctcct
*P53(human)*	ccccagccaaagaagaaacc	gcctgggcatccttgagttc
*CACNA1C*	ggctgctgaggattttcaag	acacagtgaggagggactgg
*KCNE3*	tccagagacatcctgaagagg	ggtctccgttccattggtag
*KCND3*	tgatgttttatgccgagaagg	ccatggtgactccagctctt
*KCNN1*	agccaccctctctcccagtca	aggggttgggctcgctgca
*SCN2A*	atgatgaaaatggcccaaag	ggtggcactgaatcgagaga
*SCN3A*	atgctgggctttgttatgct	ttgctcctttcccagtaagc
*SCN4A*	tccagcagggttggaatatc	tgccaatgatcttgatgagc
*SCN5A*	ccagatctctatggcaatcca	gaatcttcacagccgctctc
*SCN9A*	gatgatgaagaagccccaaa	gtggcattgaaacggaagat
*SCN10A*	acttgaaagcctgcaaccag	cactaaaccgggaaatggtc
*SCN1B*	tctaccgcctgctcttcttc	ggcagcgatcttcttgtagc
*SCN2B*	atccatctgcaggtcctcat	catctgtgctcagcttctgc
*KCNQ1*	ggccacggggactctcttc	tccgtcccgaagaacacca
*KCNA5*	ggccgaccccttcttcatc	gcagctcgaaggtgaaccag
*KCNH2*	gaggagcgcaaagtggaaatc	gccccatcctcgttcttcac
*KCNJ5*	ttctgaagggagcaggtcat	cctagaatcgccagccatag
*KCNE2*	cttgtgtgcaacccagaaga	gtcttccagcgtctgtgtga
*Gapdh(mouse)*	tggccttccgtgttcctacc	ggtcctcagtgtagcccaagatg
*GAPDH(human)*	aaggtgaaggtcggagtcaac	ggggtcattgatggcaacaata

### Electrophysiological studies

The sodium current was detected by patch-clapping on HEK293 cells. When HEK293 cells were cultured for 70–80 % confluent in 9.6 cm^2^ plates, 2 μg pcDNA-SCN5A and 500 ng pEGFP-N1 or 1 μg pcDNA-SCN5A and 1 μg pEGFP-N1-SCN3B were transfected into cells using lipofectamine2000 and Opti-Modified Eagle’s medium (OMEM). After cultured for 4–6 h, OMEM was replaced with DMEM and 1 ng/μl IL-2 was added into IL-2 treated group as well as ddH_2_O was added into control group. Cells were cultured for 48 h and GFP-positive cells were selected for electrophysiological studies.

Sodium current were recorded at room temperature (22 °C–25 °C) using a Multiclamp 700B amplifier (Axon Instruments, Sunnyvale, CA) [10]. Patch pipettes (tip resistance was 2–3MΩ) were filled with following solutions as described previously [11]: 20 mM NaCl, 130 mM CsCl, 10 mM HEPES, 10 mM EGTA, pH 7.2, with CsOH. The components of bath solution was 70 mM NaCl, 80 mM CsCl,5.4 mM KCl, 2 mM CaCl_2_, 1 mM MgCl_2_, 10 mM HEPES, 10 mM glucose, pH 7.3, with CsOH (All products were purchased from Sigma, Madison, WI, USA). Junction potential, capacitance and series resistance were automatically compensated in the whole cell configuration. The holding potential was maintained at -120 mV, and the voltage clamp were operated as described [12]. The sodium currents were filtered at 5 kHz, sampled at 50 kHz, and stored on a desktop computer with equipped with an AD converter (Digidata 1440A, Molecular Devices). All current measurements were normalized using the cell capacitance. The Clampfit 10.2 (Axon Instruments), Excel (Microsoft), and Origin 85 (Microcal Software) were used for data acquisition and analysis.

### Western blot analysis

Western blot analysis was performed to observe the expression of proteins in HL-1 cells after stimulating by mouse homologous IL-2. Cells were cultured in 9.6 cm^2^ plates and transfected as described. After 48 h, cells were harvested and incubated in ice-cold TNEN lysis buffer (in mmol/L: 50 mM Tris/HCl, pH 7.5, 150 mM NaCl, 2.0 mM EDTA, 1.0 % Nonidet P-40) with 1 mini tab of EDTA-free protease inhibitors (Roche) and 1 mmol/L PMSF (phenylmethylsulfonyl fluoride) for 30 min at 4 °C. The insoluble fraction was pelleted by centrifugation at 12,000 x g for 15 min at 4 °C. 100 μl of supernatant was mixed with 20 μl of 6X laemmli buffer (0.3 mol/L Tris–HCl, 6%SDS, 60 % glycerol, 120 mmol/L dithiothreitol (DDT) and proprietary pink tracking dye), and heated at 37 °C for 10 min. 20 μl of samples were subjected to SDS-PAGE. Proteins were transferred onto a 0.45um polyvinylidene fluoride (PVDF) membrane (Millipore) after electrophoresis. The membrane was probed with an anti-p53 mouse monoclonal antibody (abcam, PAb240) or anti-scn3b rabbit polyclonal antibody (GeneTex, GTX104440), followed by incubation with a HRP-conjugated secondary goat anti-mouse or goat anti-rabbit antibody respectively (Millipore). The protein signal was visualized by a Super Signal West Pico Chemi luminescent substrate according to the manufacturer’s instructions (Pierce Chemical Co., Rockford, Illinois, USA). Mouse GAPDH (Proteintech, 14C10) was used as loading control. Each assay was performed in triplicate and repeated at least three times.

### Statistical analysis

All the experimental data were from three independent experiments and presented as means and standard deviation (S.D.). Statistical analysis was performed with a Student’s *t*-test using SPSS version 17.0 software (SPSS, Chicago, IL, USA). Differences were considered significant when *P* value < 0.05. Multiple comparisons were applied using Bonferroni correction and Benjamini-Hochberg correction.

For analysis of the data from real-time RT-PCR analysis, we calculated the means for RQ values from the 3 wells and then compare the means from three independent experiments between two different groups with a Student’s *t*-test. For Western blot analysis, the images from three independent experiments were scanned with Quantity One 4.6.8 (Basic) (Bio-Rad, Hercules, California, USA) and quantified. The means from three independent experiments were compared between two different groups with a Student’s *t*-test.

## Results

### IL-2 up-regulated the expression of *SCN3B/Scn3b*

**Fig. 1:**
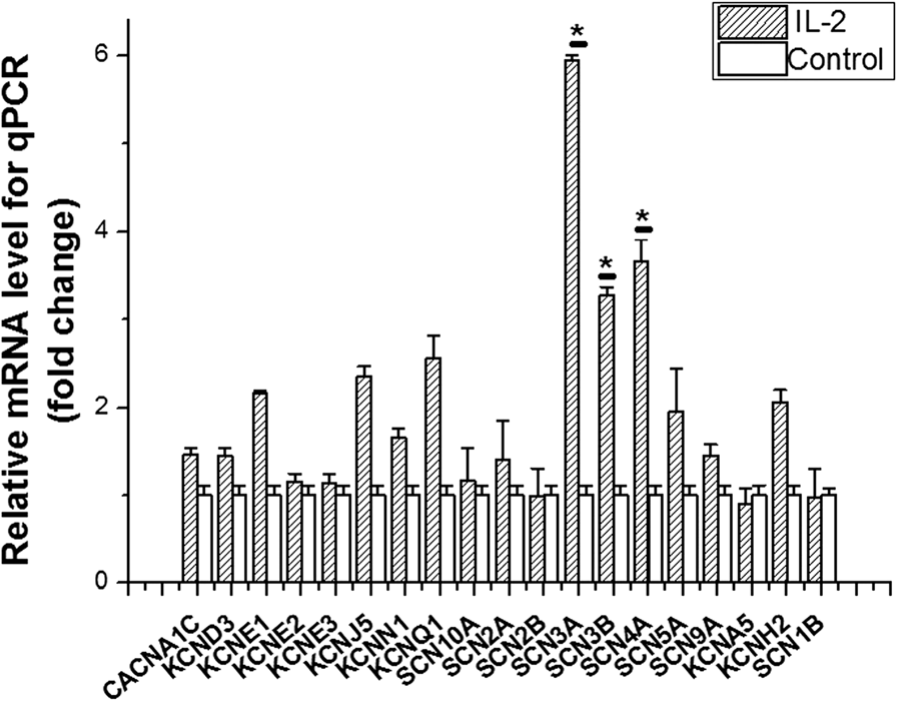
Effect of dealing with interleukin 2 (IL-2) on regulation of ion channels endogenous expressed in Human cervical carcinoma cells (HeLa cells) by quantitative real-time chain reaction (qRT-PCR) analysis. The mRNA samples were prepared from transfected HeLa cells. *GAPDH* was used as a control for normalization. *SCN3A*, *SCN3B* and *SCN4A* were up-regulated in transcription over 3-fold. *KCNE1*, *KCNJ5*, *KCNH2* and *KCNQ1* were also up-expression over 2-fold. Each experiment was performed in triplicate presented as means and standard deviation (S.D.). *p < 0.05

*SCN2A*, *SCN3A*, *SCN4A*, *SCN5A*, *SCN9A*, *SCN10A*, *SCN1B, SCN2B*, *SCN3B*, *KCNN1*, *KCNJ5*, *KCNE1, KCNE2*, *KCNE3*, *KCND3, KCNQ1, KCNA5, KCNH2* and *CACNA1C* were endogenous expressed in HeLa cell lines (Fig. 1). After treating with 1 ng/μl IL-2 (IL-2 treated groups) or ddH_2_O (control groups) for 48 h, the mRNA levels of *SCN3A*, *SCN3B* and *SCN4A* were up-regulated in IL-2 treated groups (Fig. 1) and the mRNA level of *SCN3B* was up-regulated most significantly (3.28 folds, *p* = 0.02 compared with the control groups). The mRNA levels of *KCNE1, KCNJ5, KCNH2* and *KCNQ1* were also up-expressed over 2-fold in IL-2 treated group but were not significant compared with the control groups (*p* > 0.05). The rest genes including *SCN1B, CACNA1C*, *KCND3*, *KCNE2*, *KCNE3*, *KCNN1*, *SCN10A*, *SCN2A*, *SCN5A*, *SCN9A* and *KCNA5* were up-expressed less than 2-fold. The results showed that IL-2 significantly increased the transcriptional level of several genes which encoded the subunit of sodium channels (*SCN3A*, *SCN3B* and *SCN4A*).

**Fig. 2:**
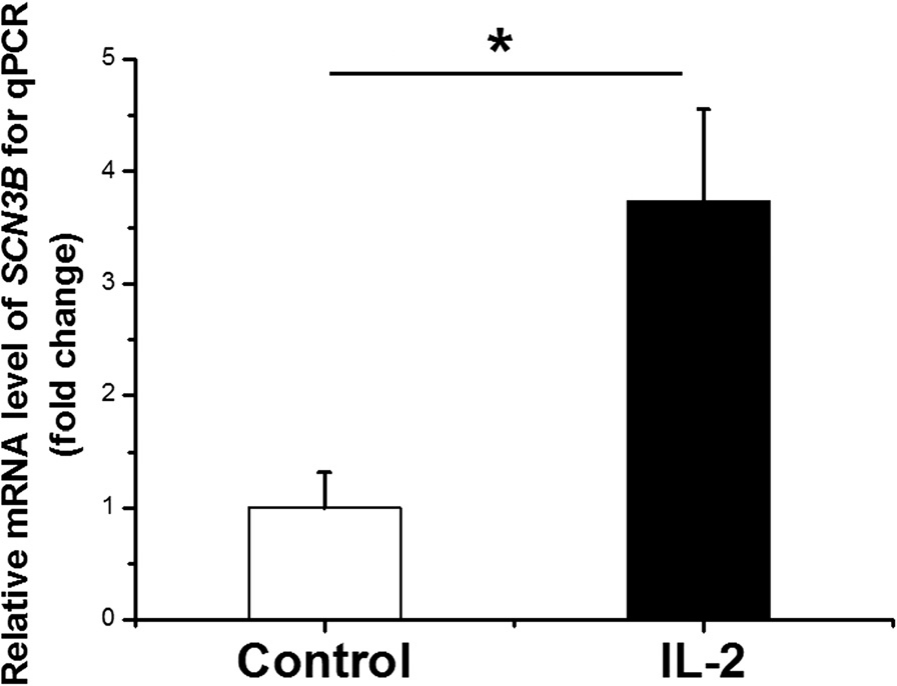
Effect of dealing with interleukin 2 (IL-2) on regulation of *Scn3b* in mouse myocardial HL-1 cells by quantitative real-time chain reaction (qRT-PCR) analysis. The mRNA samples were prepared from transfected HL-1 cells. *Gapdh* was used as a control for normalization. *Scn3b* was 3.73-fold up-regulated in transcription (*p* = 0.01). Each experiment was performed in triplicate presented as means and standard deviation (S.D.). *p < 0.05

Because *SCN3A* and *SCN4A* mostly expressed in brain and skeletal muscle respectively, while *SCN3B* expressed in myocardium, then we carried out the experiment on mouse myocardial HL-1 cells and observed that the mRNA level of *Scn3b* was still up-regulated significantly (Fig. 2. 3.73 folds, *p* = 0.01 compared with the control groups). To control of multiple comparisons, we first applied the Bonferroni correction to calculate adjusted *p* values across 19 genes, unfortunately none of genes stood out (<0.05/19). Furthermore we applied Benjamini-Hochberg correction which was less conversed, but the results was consistent with Bonferroni correction. To reduce false positive signal due to our small sample size, we further validated the top signal *SCN3B* in a second independent experiments. Consistent results were showed in HEK293 cells using RT-PCR analysis (Additional file 1: Figure S1, 1.57 folds for *SCN3B*, *p* = 0.05; 1.43 folds for *P53*, *p* = 0.02). Based on above, we think the top gene *SCN3B* is an idea candidate for further electrophysiological studies.

### IL-2 causes the gain-of-function-like effect of *SCN3B* and increases the sodium current (I_Na_)

**Fig. 3:**
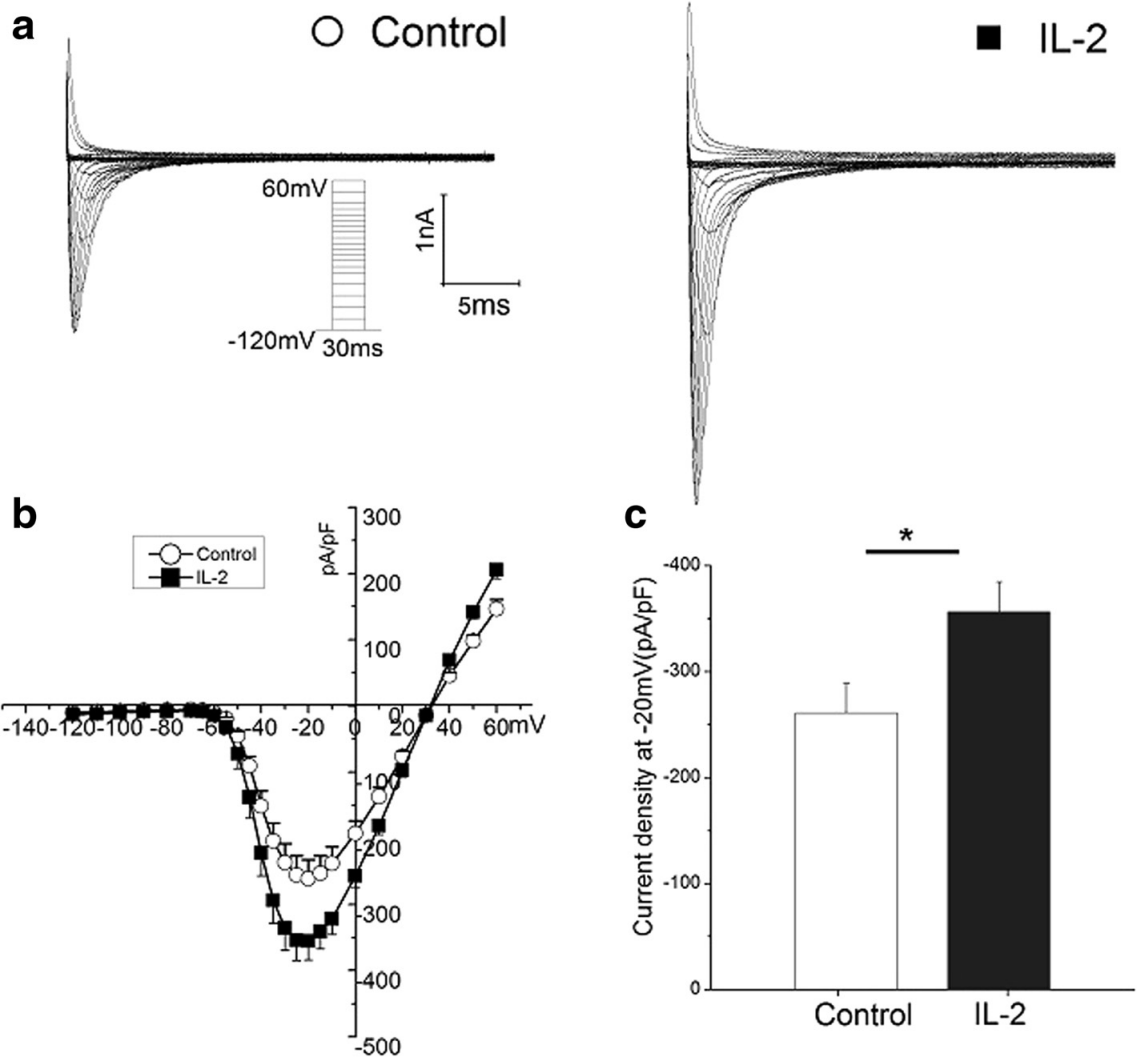
Interleukin 2 (IL-2) increased the sodium current density dependent on SCN3B. **a** Representative traces for sodium currents without (*left*) and with (*right*) treated by IL2were elicited with the current protocol depicted in the inset. **b**. I-V relation for peak sodium current Nav1.5. Average Nav1.5 sodium current density is greater by treatment of IL2 in the presence of SCN3B. **c** Histogram of sodium current densities at −20 mV. Means and SEM: Control (−260.6 and 28.1 pF/pA *n* = 17), IL2 (−355.9 and 28.3 pF/pA *n* = 21).*p < 0.05

**Fig. 4:**
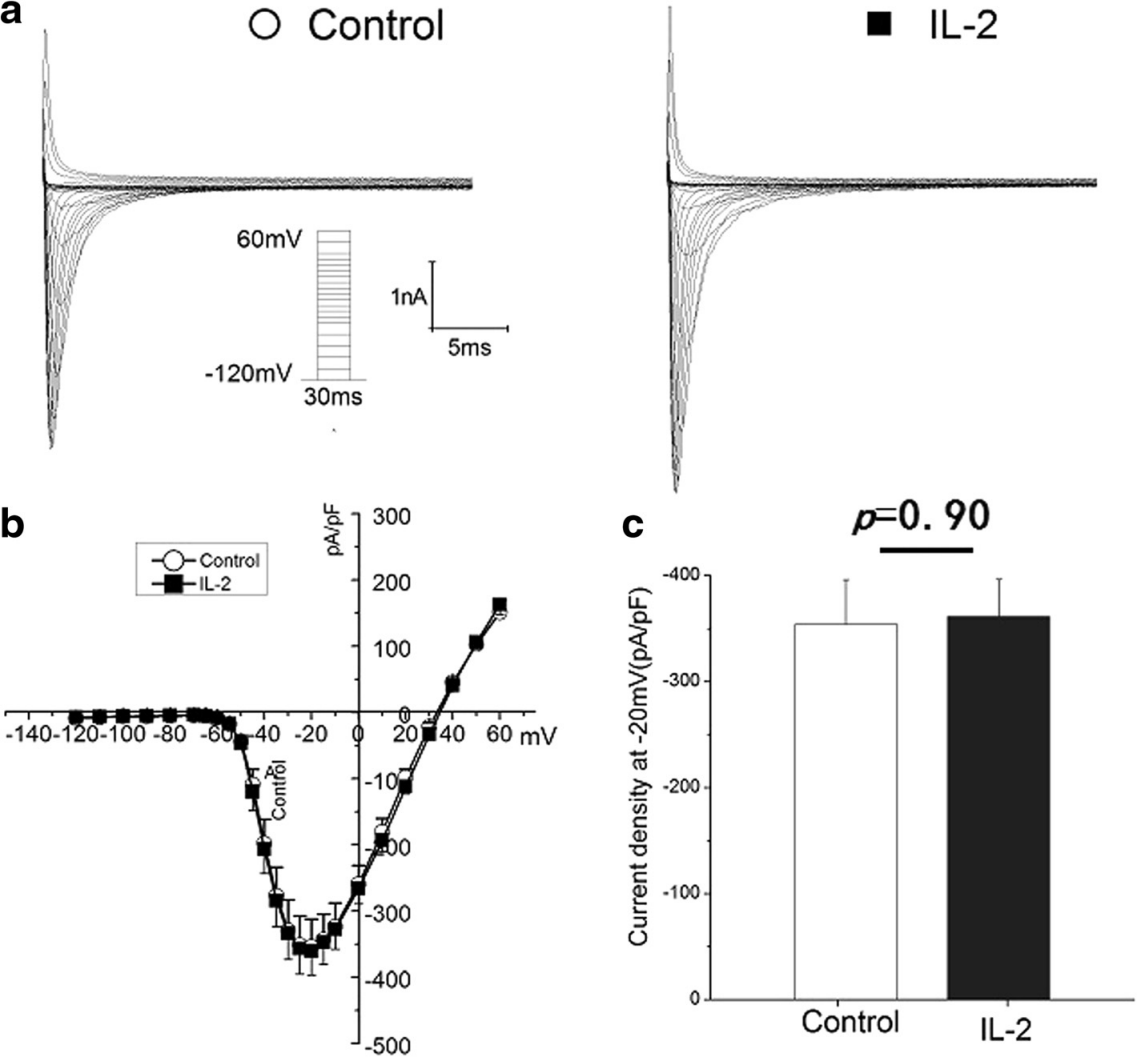
Interleukin 2 (IL-2) failed to increase the sodium current density in the absence of SCN3B. **a** Representative traces for sodium currents without (*left)* and with (*right*) treated by IL-2 were elicited with the current protocol depicted in the inset. **b**. I-V relation for peak sodium current Nav1.5. Average Nav1.5 sodium current density is similar between with and without IL-2 in the absence of SCN3B. **c** Histogram of sodium current densities at −20 mV. Means and SEM: Control (−354.7 and 41.7 pF/pA *n* = 20), IL2 (−361.0 and 35.3 pF/pA *n* = 17). NS = Not Significant

The sodium current was commonly analyzed by patch-clapping of HEK293 cells. In order to determine if IL-2 affect the sodium current via increasing the expression level of *SCN3B*, we transfected both *SCN3B* and *SCN5A* or only *SCN5A* into HEK293 cells. The sodium current density (expressed as peak current normalized to cell capacitance, pA/pF) across the range of test potentials was significantly increased after treated by IL-2 (Fig. 3a and b). Comparing to untreated cells, the sodium current density across the range of test potentials was increased 1.4 folds in the cells which were transfected both *SCN3B* and *SCN5A* after IL-2 treated (Fig. 3c, *p* = 0.02). While IL-2 failed to affect the sodium currents in cells expressing the *SCN5A* (Fig. 4, *p* = 0.90) alone.

### IL-2 actively regulates the expression of *p53* and *scn3b* in mouse myocardial cells

**Fig. 5:**
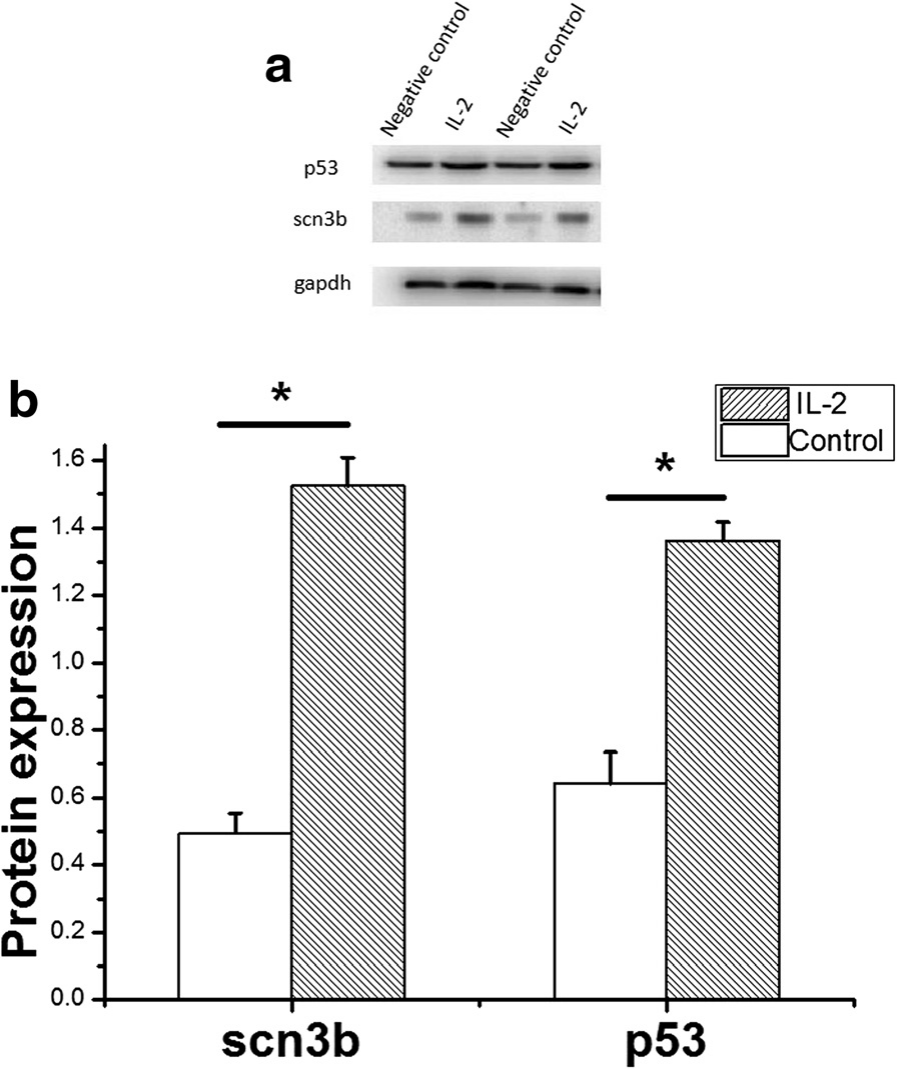
The expression of *p53* and *scn3b* in HL-1 cells induced by Interleukin 2 (IL-2) by Western blot analysis. The protein samples were prepared from transfected HL-1 cells. *Gapdh* was used as a control for normalization. *P53* and *Scn3b* were significantly increased induced by IL-2 compared with negative control. The images of Western blot analysis shown in (**a**) were scanned, quantified and plotted in (**b**). Data is shown as means and SD

Because IL-2 actively regulates the expression of *p53* in T cells [13] and p53 induces the expression of *SCN3B* in proapoptotic cells [14]. Then we performed the Western blot analysis to identify the effect of IL-2 on the expression of *p53* and *SCN3B* in mouse myocardial HL-1 cells. The results showed that IL-2 significantly increased the expression level of *p53* and *scn3b* (Fig. 5, 2.1 folds, *p* = 0.02 for *p53*; 3.1 folds, *p* = 0.02 for *scn3b*) in HL-1 cells.

## Discussion

In the present study, we observed that pro-inflammatory cytokine IL-2 can affect the expression of *SCN3B*, which encodes the β3 subunit for sodium channels [15], and increase the sodium current by the its effect on *SCN3B* for the first time.

IL-2 is a potent inducer for T cell proliferation as well as Th1 and Th2 differentiation and has been demonstrated may act as a key factor for many cardiovascular diseases and arrhythmia such as AF [2]. Previous studies showed that IL-2 could increase activity of sarcoplasmic reticulum Ca^2+^-ATPase but decrease its sensitivity to calcium in rat cardiomyocytes and IL-2 affected Ca^2+^ not from reduced activity of the L-type calcium channel [16]. Similar to above results, we observed that IL-2 didn’t affect the mRNA level of the *CACNA1C*, which encode the α1C subunit of L-type calcium channel. However, IL-2 can increase the mRNA level of genes which encodes the subunit for sodium channels, especially *SCN3B.SCN3B* encodes the β3 subunit for sodium channels and in 2009, Dan Hu, *et al.* detected a missense mutation (L10P) in exon 1 of *SCN3B* and provided the mutation led to an 82.6 % decrease in peak sodium current density, accelerated inactivation, slowed reactivation, a −9.6 mV shift of half-inactivation voltage and clinical manifestation of a Brugada syndrome, which is a disorder characterized by malignant ventricular arrhythmia and sudden death [17]. Subsequently, Carmen R. V., et al. identified a mutation V54G in *SCN3B* in a 20-year-old male who suffered ventricular fibrillation (VF) and observed that mutation V54G in *SCN3B* decrease peak sodium current significantly.[18]. The same year, we identified a mutation A130V in *SCN3B* dramatically decreased the cardiac sodium current density and led to AF by a dominant negative mechanism, in which the mutant protein negated or counteracted with the function of wild type *SCN3B* [19]. Then Morten S. Olesen *et al.* sequenced coding sequence of *SCN3B* in 192 unrelated AF patients and found three non-synonymous mutations in *SCN3B*, which led to loss of function in the sodium current by affecting biophysical parameters of conducted sodium current [20]. Furthermore, *SCN3B* knockout mice developed AF under atrial burst pacing protocols [21]. These results suggested that *SCN3B* may be critical to the pathogenesis of arrhythmia. Mutations in *SCN3B* impaired intracellular trafficking of sodium channel Nav1.5 to plasma membranes, decreased the density of cardiac sodium currents and play role in the mechanism of arrhythmia [22]. In present study, we observed that high expression level of the *SCN3B* which induced by IL-2 can increase the sodium current density, these results suggested that increased serum level of IL-2 can affect various cardiac arrhythmias [6, 7] by its effect on the *SCN3B* and sodium current density. In the present study, the increased expression of *scn3b* was associated with the expression level of *p53*, which was induced by IL-2 significantly. The results indicated that the IL-2 related expression of *SCN3B* may be regulated via the p53 pathway [13] and further studies are needed to reveal the exact mechanism.

## Conclusions

In conclusion, the present study suggested that IL-2, a pro-inflammatory cytokine, may play role in the arrhythmia by its affection on *SCN3B* and sodium current density.

### Limitation

In the present study, we observed the effect of IL-2 on the transcription of ion channel genes including *SCN2A*, *SCN3A*, *SCN4A*, *SCN5A*, *SCN9A*, *SCN10A*, *SCN1B*, *SCN2B*, *SCN3B*, *KCNN1*, *KCNJ5*, *KCNE1, KCNE2*, *KCNE3*, *KCND3, KCNQ1, KCNA5, KCNH2* and *CACNA1C*. Based on the above findings, we speculate that the top gene *SCN3B* might be a good candidate. Nevertheless, it never rules out the possibility of false positive signal because it didn’t stand out after multiple comparisons. Further parallel and independent studies with larger sample size are warranted.

## Additional file

Additional file 1: Figure S1. Effect of dealing with interleukin 2 (IL-2) on regulation of *SCN3B* and *P53* in HEK293 cells by quantitative real-time chain reaction (qRT-PCR) analysis. The mRNA samples were prepared from transfected HEK293 cells. *GAPDH* was used as a control for normalization. *SCN3B* was 3.1-fold up-regulated in transcription (*p* = 0.02) and *P53* was 2.1-fold up-regulated in transcription (*p* = 0.05). Each experiment was performed in triplicate presented as means and standard deviation (S.D.). **p* < 0.05. (JPEG 106 kb)

## Abbreviations

AF: Atrial fibrillation
IL-2: Interleukin 2
CRP: C-reactive protein
hsCRP: High sensitivity C reactive protein
TNF-α: Pro-inflammatory factors tumor necrosis factor α
IL-1: Interleukin 1
MCP-1: Monocyte chemo attractant protein 1
VT: Ventricular tachycardia (VT)
CABG: Cardiopulmonary bypass graft
PAF: Postoperative AF
