# Supplementary figures and images for: Regulation of SCN3B/scn3b by Interleukin 2 (IL-2): IL-2 modulates SCN3B/scn3b transcript expression and increases sodium current in myocardial cells
Source: BMC Cardiovasc Disord. 2016 Jan 4;16:1. doi: 10.1186/s12872-015-0179-x (PMC4700781; doi:10.1186/s12872-015-0179-x)

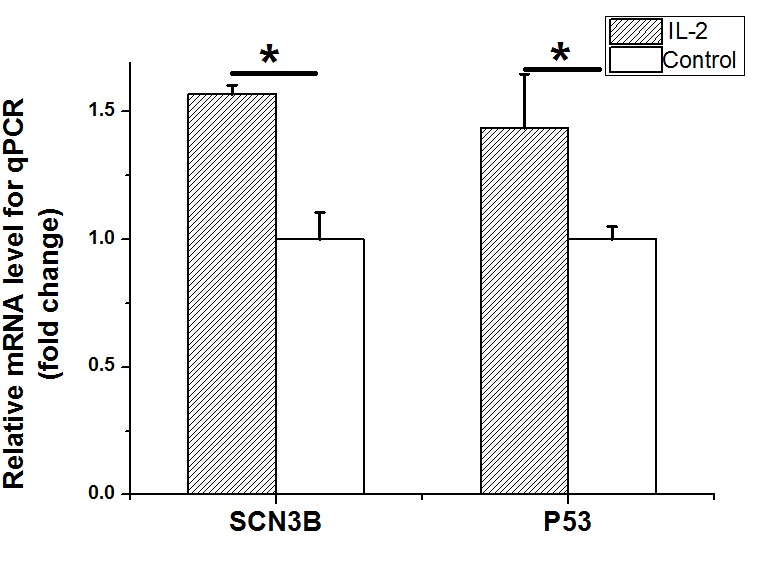

Supplement: Additional file 1: Figure S1. — Effect of dealing with interleukin 2 (IL-2) on regulation of SCN3B and P53 in HEK293 cells by quantitative real-time chain reaction (qRT-PCR) analysis. The mRNA samples were prepared from transfected HEK293 cells. GAPDH was used as a control for normalization. SCN3B was 3.1-fold up-regulated in transcription (p = 0.02) and P53 was 2.1-fold up-regulated in transcription (p = 0.05). Each experiment was performed in triplicate presented as means and standard deviation (S.D.). *p < 0.05. (JPEG 106 kb) [file 12872_2015_179_MOESM1_ESM.jpeg]
